# Involvement of the maxillary sinus ostium (MSO) in the edematous processes after sinus floor augmentation: a cone-beam computed tomographic study

**DOI:** 10.1186/s40729-020-00233-7

**Published:** 2020-08-03

**Authors:** Shigeru Sakuma, Mauro Ferri, Hideki Imai, Natalia Fortich Mesa, Daniel José Blanco Victorio, Karol Alí Apaza Alccayhuaman, Daniele Botticelli

**Affiliations:** 1 ARDEC Academy, Viale Giovanni Pascoli 67, 47923 Rimini, Italy; 2 ARDEC Foundation, Cartagena, de Indias, Colombia; 3 Department of Oral Implantology, Osaka Dental University, Osaka, Japan; 4 Corporación Universitária Rafael Núñez, Cartagena de Indias, Colombia; 5 Universidad Peruana Cayetano Heredia, Lima, Perú; 6 Department of Oral Biology, University Clinic of Dentistry, Medical University of Vienna, Vienna, Austria

**Keywords:** Maxillary sinus, Cone-beam computed tomography, Sinus mucosa, Schneiderian membrane, Maxillary sinus ostium

## Abstract

**Background:**

After sinus floor augmentation, a thickening of the sinus mucosa has been described. The aim of the present study was to evaluate the involvement of the maxillary sinus ostium in the edematous processes after a sinus floor augmentation procedure.

**Methods:**

Seventy-two cone-beam computerized tomographies (CBSTs) were taken before sinus floor augmentation and after 1 week and 9 months from surgery and analyses. Sinus mucosa thickness and area, ostium diameter and patency, and extension of the post-surgical transient mucosal thickening in relation to the ostium were evaluated on the CBCTs for all three periods. The term “virtual” when referring to sinus mucosa thickness and area was introduced because of the edema and bleeding that both contributed to a transient thickening and additional elevation of the sinus mucosa.

**Results:**

The mean virtual thickness of the sinus mucosa was 2.7 ± 4.0 mm, 7.7 ± 7.1 mm, 1.7 ± 2.0 mm before surgery, and after 1 week and 9 months. The virtual mucosa area was 37.2 ± 52.5 mm^2^, 184.5 ± 153.8 mm^2^, and 34.0 ± 50.7 mm^2^. The ostium diameter at the three periods evaluated was 1.8 ± 0.5 mm, 1.1 ± 0.6 mm, 1.5 ± 0.8 mm, respectively. Three infundibula (4.2%) were found out of patency before surgery while this number increased to 14 (19.4%) after 1 week. Nine months after surgery, only one infundibulum (1.4%) was out of patency, however, without presenting signs of sinus pathologies. The extension of the mucosal edema on the palatal sinus was reduced after 9 months of healing.

**Conclusions:**

One week after sinus floor augmentation, the maxillary sinus mucosa increased in dimensions and in several cases involved the ostium, reducing its diameter and producing a transient loss of patency. After 9 months of healing, the initial conditions were recovered.

## Introduction

The loss of the posterior maxillary teeth often results to insufficient bone volume owing to the resorption of the alveolar bone and the pneumatization of the maxillary sinus. Such conditions frequently do not allow to perform an oral rehabilitation of the region by means of implants. In order to increase the bone volume to make the installation possible, sinus floor elevation procedures have been proposed [1]. One of the most used and predictable techniques includes the sinus floor augmentation through an osteotomy in the lateral wall of the sinus [2, 3].

Nevertheless, the bleeding within the elevated space and the inflammatory phenomena (edema) associated with these procedures result in a transient additional elevation of the Schneiderian membrane besides that obtained with the filler material [4–11]. The detachment of the sinus mucosa from its bed, together with the edema and bleeding that follows the surgical intervention, also carries a risk of damage to the ciliated epithelium that might impair the mucociliary clearance [12–14]. This transient edema and bleeding might also lead to a reduction of the patency of the ostium that might predispose to a development of sinusitis [15].

Volumetric changes after the elevation of the sinus floor using different types of filler materials were evaluated using cone-beam computerized tomographies (CBCTs) [7, 9–11, 15, 16]. In those studies, the changes in the thickness of the sinus mucosa were also analyzed. In a clinical study, the patency of the maxillary sinus ostium (MSO) was reported [15]. However, no evaluations were performed about the extension of the edema towards the MSO, the level of the involvement of the MSO, and the outcome after the surgical treatment.

Hence, the aim of the present study was to evaluate the involvement of the maxillary sinus ostium in the edematous processes after a sinus floor augmentation procedure.

## Material and methods

Informed consent was obtained from all participants at the time of surgery. The present observational retrospective study followed the STROBE checklist and was approved by the Ethical Committee of the University Corporation Rafael Núñez, Cartagena de Indias, Colombia (protocol # CURN-0002-CE282020). The present study has been carried out in accordance with The Code of Ethics of the World Medical Association (Declaration of Helsinki) for experiments involving humans.

### Participants

The eligibility criteria were the following: (i) patients that received sinus floor augmentation; (ii) availability of a CBCT before sinus floor augmentation, and after 1 week and 9 months; and (ii) anatomical structures to be analyzed clearly visible and measurable for all CBCTs.

Patients’ treatments were performed at the University Corporation Rafael Núñez, Cartagena de Indias, Colombia.

### CBCT imaging procedures

Three cone-beam computed tomographies (CBCTs) were taken in the same radiological specialistic center before sinus floor augmentation (baseline T0), after 1 week (T1w) and after 9 months (T9m) from surgery, respectively. A 3D Accuitomo 170 Tomograph (J Morita Corporation, Kyoto, Japan) was used. The CBCT images were recorded at 80 kV and 8 mA, FOV 77.125;77.125;74.000. The 3D reconstruction was performed with slices at an interval of 1.0 mm with a basic voxel size of 0.125 mm.

### Data source and measurements on the CBCT images

**Fig. 1:**
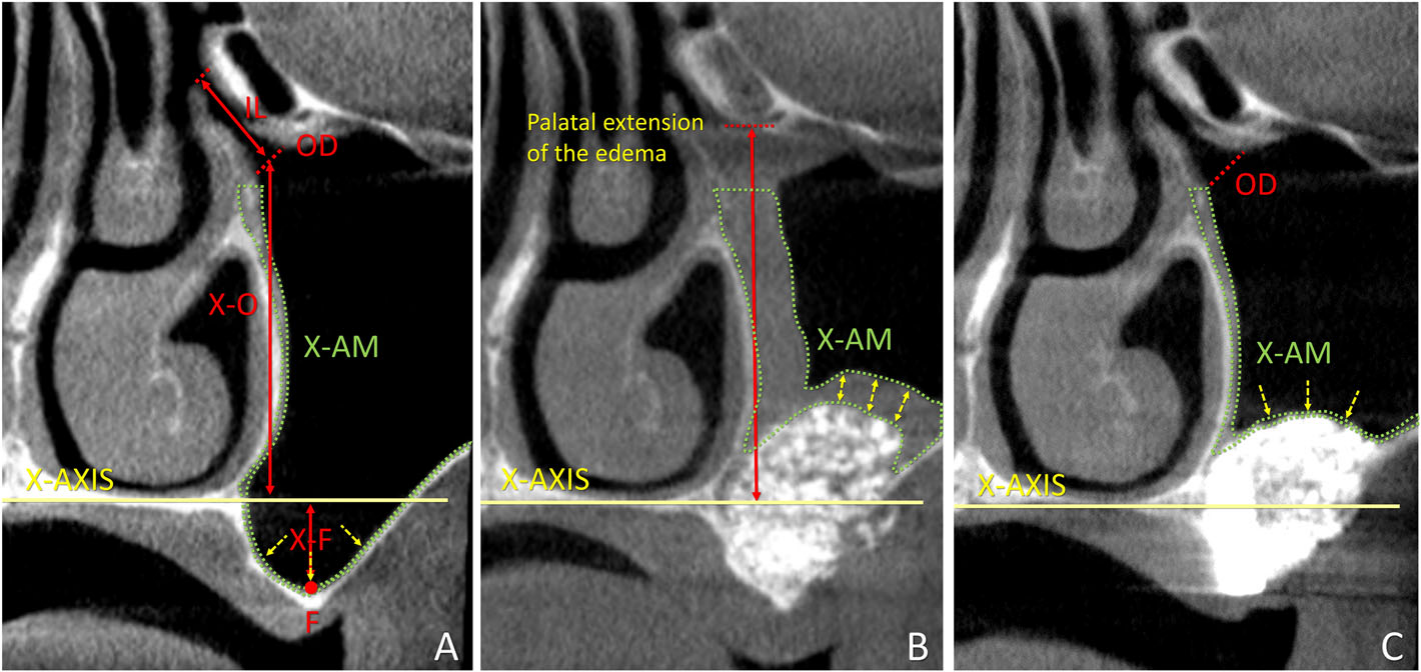
Coronal view of tomographic images of a maxillary sinus before surgery (**a**), and after 1 week (**b**) and 9 months (**c**). *X*-axis, nose floor in the coronal view; X-AM, area of the sinus mucosa (delimited by dotted green lines); IL, infundibulum length; OD, maxillary sinus ostium diameter; F, sinus floor; X-F, distance between *X*-axis and F; X-O, distance between *X*-axis and maxillary sinus ostium. Yellow arrows, positions of the measurements of the thickness of the mucosa

**Fig. 2:**
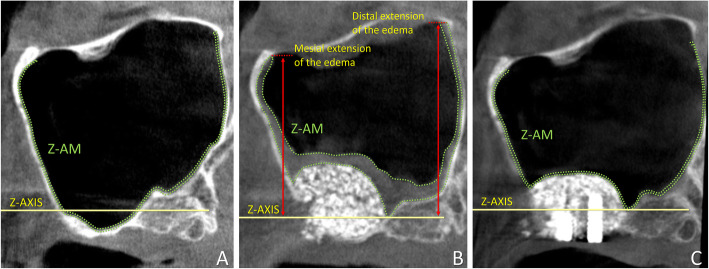
Lateral view of tomographic images of a maxillary sinus before surgery (**a**), and after 1 week (**b**) and 9 months (**c**). *Z*-axis, nose floor in the lateral view; Z-AM, area of the sinus mucosa (delimited by dotted green lines); mesial extension and distal extension, the vertical extension of the edematous mucosa at the mesial and distal aspects in relation to *Z*-axis, respectively

The CBCT analyses were made using the i-Dixel 2.0 software (J. Morita Corporation, Kyoto, Japan). The nose floor was selected as a reference plane for both the coronal (*X*-axis; Fig. 1), and the lateral views (*Z*-axis; Fig. 2). A line crossing the anterior nasal spine and the nasal septum was used as a vertical reference plane in the coronal view [9, 10, 17].

Various parameters (Table 1) were evaluated in the section intersecting the MSO in the coronal view (Fig. 1) and in lateral view (Fig. 2). The virtual sinus mucosa thickness and area included the real sinus mucosa and the clot/edema present after surgery and possible pathologies present before and after surgery. The evaluations were performed in the coronal section (Y slice image) corresponding to the center of the ostium. The thickness of the mucosa was measured in three positions at the baseline, i.e., at the bottom of the sinus and in a middle position between the bottom on the sinus and the *X*-axis, both at the medial and lateral walls. In the 1-week CBCTs and 9-month CBCTs, the thickness was measured in three positions about equidistant between the two angles formed by the sinus mucosa lining on the elevate space and that on the medial and lateral sinus walls (Fig. 1). The mean values of the three measurements were recorded for each period. The patency of the infundibulum was evaluated moving back and forth the field of view on the *Y*-axis. A similar procedure was performed for the evaluation of accessory ostia.

**Table 1:** Parameters evaluated on the CBCTs at the various periods

T0 - Coronal view	Distance between the maxillary sinus ostium (MSO) to the *X*-axis.
	Distance between MSO to the floor of the maxillary sinus.
	The maxillary infundibulum length, included between the maxillary sinus ostium and the hiatus semilunaris.
	The diameter and position of accessory maxillary ostia (ASO).
T0, T1w, T9m - Coronal view	The diameter of the MSO.
	Patency of the infundibulum evaluated as the number of obstructions.
	Virtual mucosa thickness evaluated as the distance between bone/biomaterial and the outer contour of the sinus mucosa.
	Virtual area of the sinus mucosa. The limits of the areas measured were defined by the extension of the edema in the 1-week CBCT. In the palatal aspect, the maximum limit of the measurements of the edema was placed at the base of the ostium. In the lateral aspect, this limit was defined by the field of view that cut the image in this region.
T1w, T9m - Coronal view	Distance between the vertical extension of the edematous mucosa and the MSO.
T0, T1w, T9m - Lateral view	Virtual area of the sinus mucosa. The limits of the areas measured were defined by the extension of the edema in the 1-week CBCT. The maximum limit of the measurements of the edema was the intersection of the superior wall with the mesial and distal sinus walls, respectively.
T1w, T9m - Lateral view	The vertical extension of the edematous mucosa at the mesial and distal aspects in relation to *Z*-axis.

*T0* before surgery, *T1w* after 1 week, *T9m* after 9 months, *MSO* maxillary sinus ostium

### Control of biases and statistical methods for data analysis

The CBCT assessments were performed by a calibrated examiner (KAAA) with an intra-examiner coefficient *k* > 0.9 for all variables. The intra-reproducibility of the measurements was facilitated using well-defined landmarks for all patients [9, 10, 17]. Two measurements were made for each variable, and mean values were calculated. Mean values, standard deviations, maximum, and minimum values were subsequently calculated for each parameter.

### Statistical analysis

Descriptive statistical analyzes were performed using Stata 15.0 software (Stata Corporation, College Station, TX, USA) and R version 4.0.0 (2020-04-24)—“Arbor Day” Copyright (C) 2020 The R Foundation for Statistical Computing. The data were subjected to descriptive statistical analysis calculating measures of the central tendency and dispersion. The normality and homogeneity of the variables were evaluated using the Shapiro-Wilk and Bartlett’s tests, respectively. In non-compliance with the assumptions, it was decided to use non-parametric tests such as the Friedman test for repeated measures in three times and as post hoc the Nemenyi test for multiple comparisons to identify pairs with significant differences. A significance level of 5% (*p* < 0.05) was considered for the statistical results of the tests.

A logistic regression to study the association among gender, age, smoke, side, type of edentulism, ostium position, and the patency of the infundibulum after surgery was performed.

## Results

In the present observational retrospective study, the CBCTs of seventy-two sinuses, from fifty-nine mulatto patients (Table 2), have been evaluated in three different periods (two-hundred sixteen CBCT in total): before sinus floor augmentation (T0), and after 1 week (T1w) and 9 months (T9m). The visits, the surgical procedures, and the follow-up were performed in the clinic of the University Corporation Rafael Núñez of Cartagena de Indias, Colombia, from August 2015 to March 2018.

**Table 2:** Demographic and clinical data. Patients, *n* = 59; sinuses, *n* = 72

Gender	Age (years)	Smokers	Side	Type of edentulism	Ostium position
39 females; 20 males	53.3 ± 9.1	Name	42 right; 30 left	58 partial, 14 total	21 PM1, 31 PM2, 20 M1

### Clinical report

Fifty-nine patients, 39 females and 20 males with a mean age of 53.3 ± 9.1 years, were included in the study (Table 2). The CBCTs of forty right and thirty-two left maxillary sinuses treated for floor augmentation were analyzed. All access windows were prepared grinding the bone with a diamond insert (SFS 109 029, Komet-Brasseler-GmbH, Germany) mounted on a sonic-air surgical instrument (Sonosurgery® TKD, Calenzano, Fi - Italy). Two different xenografts were used as filler material: a collagenated cortico-cancellous porcine bone (OsteoBiol Gen-Os®, 250–1000 μm, Tecnoss, Giaveno, Italy) or a bovine cancellous bone (Cerabone®, 1.0-2.0 mm, Botiss Biomaterials GmbH, Zossen, Germany). Six perforations of the sinus mucosa occurred during surgery; all protected with a collagen membrane. No not-expected complications, such as infections or relevant loss of biomaterial, were observed during the visits, and no complaints were reported by the patients.

### CBCT imaging evaluation—coronal view (Tables 3 and 4)

In the coronal view, the mean distance between the floor and the maxillary sinus ostium (MSO) was 33.3 ± 3.6 mm and the mean length of the infundibulum was 8.8 ± 1.8 mm. The mean diameter of the MSO was at T0 1.8 ± 0.5 mm, at T1w 1.1 ± 0.6 mm, and at T9m 1.5 ± 0.8 mm. A loss of patency in the infundibulum was found in three cases before surgery at T0 (4.2%), in fourteen cases at T1w (19.4%), and in one case at T9m (1.4%). In this last case, it was not possible to find a clear patency for the entire length of the infundibulum throughout the MSO. Moreover, no accessory maxillary sinus ostia were found in this patient. Nevertheless, no pathological signs were found within the sinus, and the mucosa presented normal dimensions. No complaints were reported by the patient. The differences between the three times were statistically significant (*p* value = 0.0001) as evaluated by the Friedman test. Performing post hoc analysis by the Nemenyi test, it was observed that there were significant differences between T0 and T1w, between T0 and T9m, and between T0 and T9m.

**Table 3:** Radiographic anatomical data in the coronal view taken at the different periods of healing. Data in millimeters or square millimeters only for the mucosa area

	Mean	SD	IQR	Min-max	p50**	p*
MSO diameter						
T0	1.83	0.54	0.54	0.94-4.6	1.77^a^	0.0001*
T1w	1.07	0.65	0.71	0-2.64	1.17^a^	
T9m	1.55	0.77	0.52	0-6.42	1.40^a^	
Virtual mucosa thickness						
T0	2.74	3.97	1.98	0.31-19.18	1.38^a^	0.0001*
T1w	7.70	7.06	6.51	0.46-31.17	5.38^ab^	
T9m	1.65	1.98	1.38	0.31-11.42	0.98^b^	
Virtual mucosa area						
T0	37.18	52.52	28.3	3.8-280.8	16.76^a^	0.0001*
T1w	184.49	153.78	239.51	5.34-581.38	140.8^ab^	
T9m	34.03	50.72	23.45	5.73-283.01	15.52^b^	

CBCTs taken at T0 (before surgery), at T1w (1 week after surgery), and at T9m

*SD* standard deviation, *IQR* interquartile range, *MSO* maxillary sinus ostium

*Friedman test, *p* < 0.05 significant

**Nemenyi test post hoc: equal letters significant differences *p* < 0.05

**Table 4:** Radiographic changes over the three periods evaluated in the coronal view. Data in millimeters or square millimeters only for the virtual mucosa area

		Δ Ostium diameter	Δ Virtual mucosa thickness	Δ Virtual mucosa area	Δ Distance between ostium and edema
**Mean values** ± SD Minimum; maximum	T1w–T0	**− 0.8** ± 0.7 **−** 2.1; 1.4	**5.0** ± 7.1 **−** 4.0; 29.6	**147.3** ± 154.3 **−** 33.5; 555.8	–
	T9m–T1w	**0.5** ± 0.7 **−** 0.6; 3.8	**− 6.0** ± 7.0 **−** 30.6; 5.0	**− 150.5** ± 150.9 **−** 561.0; 36.8	**12.3** ± 9.8 **−** 0.1; 31.6
	T9m–T0	**− 0.3** ± 0.9 **−** 1.7; 5.2	**− 1.1** ± 3.8 **−** 14.1; 10.8	**− 3.1** ± 52.3 **−** 93.2; 274.3	–

CBCTs taken at T0 (before surgery), at T1w (1 week after surgery), and at T9m

*SD* standard deviation

The mean virtual sinus mucosa thickness increased from 2.7 ± 4.0 mm at T0 to 7.7 ± 7.1 mm at T1w and decrease to 1.7 ± 2.0 mm at T9m. The differences between the three times were statistically significant (*p* value = 0.0001) as evaluated by the Friedman test. Performing post hoc analysis by Nemenyi test, it was observed that there were significant differences between T0 and T1w and between T1w and T9m.

The virtual area of the sinus mucosa was 37.1 ± 52.5 mm^2^ at T0 and increased to 184.5 ± 153.8 mm^2^ at T1w and decreased to 34.0 ± 50.7 mm^2^ at T9m. The distance between the MSO and the edematous palatal mucosa was at T1w 10.3 ± 9.5 mm and at T9m 22.6 ± 7.1 mm. The differences between the three times were statistically significant (*p* value = 0.0001) as evaluated by the Friedman test. Performing post hoc analysis by Nemenyi test, it was observed that there were significant differences between T0 and T1w and between T1w and T9m.

Data categorized for the type of edentulism and gender are reported in Table 5.

**Table 5:** Radiographic anatomical data in the coronal view divided by gender and type of edentulism. Data in millimeters

		Floor to MSO	MSO diameter			Virtual mucosa thickness		
			TO	T1w	T9m	TO	T1w	T9m
**Mean values** ± SD	Female	**33.2** ± 3.3	**1.8** ± 0.4	**1.0** ± 0.6	**1.4** ± 0.5	**2.9** ± 4.0	**6.0** ± 4.6	**1.7** ± 2.2
	Male	**33.5** ± 4.2	**1.8** ± 0.7	**1.2** ± 0.7	**1.9** ± 1.1	**2.4** ± 4.0	**10.6** ± 9.5	**1.5** ± 1.4
	Partial	**33.6** ± 3.4	**1.8** ± 0.6	**1.1** ± 0.6	**1.5** ± 0.8	**3.0** ± 4.4	**7.9** ± 6.3	**1.7** ± 2.2
	Total	**32.2** ± 4.2	**1.8** ± 0.5	**1.1** ± 0.7	**1.6** ± 0.4	**1.8** ± 1.3	**6.9** ± 10.0	**1.3** ± 0.9

*SD* standard deviation, *MSO* maxillary sinus ostium, *T0* before surgery, *T1w* 1 week after surgery, *T9m* after 9 months after surgery

Accessory maxillary ostia (AMO) were found in seven sinuses (three females and four male patients) presenting a mean diameter of 1.9 ± 0.5 mm, slightly larger than that of the main ostia in the same patients (1.7 ± 0.53 mm). These AMOs were positioned posteriorly to the primary MSO of about 10.9 ± 2.2 mm, at a distance from the *X*-axis of 18.3 ± 4.0 mm, a value closer to the *X*-axis compared to the primary MSO in the same sinuses (26.0 ± 3.1 mm). The difference was statistically significant (*p* = 0.018).

### CBCT imaging evaluation—lateral view (Table 6)

The virtual mucosal area was 61.8 ± 77.9 mm^2^. The area increased to 289.5 ± 240.3 mm^2^ at T1w and decreased to 59.2 ± 89.3 mm^2^ at T9m. The differences between the three times were statistically significant (*p* value = 0.0001) as evaluated by the Friedman test. Performing post hoc analysis by Nemenyi test, it was observed that there were significant differences between T0 and T1w and between T1w and T9m. The vertical extension of the mucosal edema at T1w above (−) or below (−) the *Z*-axis was 11.9 ± 9.7 mm at the mesial aspect and 12.4 ± 9.9 mm at the distal aspect. At T9m, these distances were 2.2 ± 5.5 mm and 2.0 ± 5.4 mm, respectively. The differences between the two times were statistically significant (*p* value = 0.0001) as evaluated by the Wilcoxon test.

**Table 6:** Radiographic anatomical data in the lateral view taken at the different periods of healing. Data in millimeters or square millimeters only for the virtual mucosa area

	Mean	SD	IQR	Min-max	p50**	p*
Virtual mucosa area						
T0	61.75	77.87	61.29	7.33–397.71	24.25^a^	0.0001*
T1w	289.54	240.34	380.18	7.31–970.39	214.86^ab^	
T9m	59.16	89.26	46.85	8.8–575.94	25.08^b^	
Mesial vertical extension above (+) or below (−) the *Z*-axis						
T1w	11.87	9.72	17.77	− 5.73–30.02	12.23	0.0001***
T9m	2.194	5.49	8.02	− 6.75–1839	0	
Distal vertical extension above (+) or below (−) the *Z*-axis						
T1w	12.38	9.87	15.12	− 7.03–36.03	11.67	0.0001***
T9m	2.032	5.40	5.38	− 7.49–19.03	0	
T1w	10.34	9.52	16.21	− 1.71–30.28	8.66	0.0001***
T9m	22.64	7.16	9.80	2.03–34.65	24.44	

*SD* standard deviation, *IQR* interquartile range CBCTs taken at T0 (before surgery), at T1w (1 week after surgery), and at T9m (9 months after surgery)

*Friedman test, *p* < 0.05 significant

**Nemenyi test post hoc: equal letters significant differences *p* < 0.05

***Wilcoxon test *p* < 0.05 significant

No associations were found among gender, age, smoke, side, type of edentulism, ostium position, and the patency of the infundibulum at T0, T1w, and T9m (Table 7).

**Table 7:** Multiple logistic regression patency of the factors age and gender

Patency T0	*B*	SE	*Z* value	*p* value	[95% CI]	Odds ratio
Gender	− 0.52	1.38	− 0.37	0.71	− 3.21–2.18	0.59
Age	0.06	0.08	0.71	0.48	− 0.10–0.22	1.06
Intercept	− 6.36	4.78	− 1.33	0.18	− 15.74–3.01	0.001
**Patency T1w**						
Gender	− 0.42	0.69	− 0.61	0.541	− 1.76–0.92	0.66
Age	− 0.0007	0.04	− 0.02	0.984	− .071–0.07	0.99
Intercept	− 1.24	1.98	− 0.63	0.530	− 5.11–2.63	0.28
**Patency T9m**						
Gender	0					1
Age	− 0.09	.12	− 0.77	0.444	− .34–0.34	0.91
Intercept	1.026	6.041	0.17	0.87	− 10.81–10.81	2.79

*B* estimated logistic coefficient, *SE* standard error of the coefficient

## Discussion

The aim of the present study was to evaluate the involvement of the maxillary sinus ostium (MSO) in the edematous processes after a sinus floor augmentation procedure.

These edematous processes contributed to an increase of the mean virtual mucosa thickness from 2.7 mm, registered before surgery, to 7.7 mm after 1 week. Nine months after floor augmentation, the thickness decreased to 1.7 mm.

The initial thickness of the virtual sinus mucosa was higher compared to the 2 mm in thickness reported in another clinical study on patients scheduled for sinus floor augmentation [18]. Nevertheless, in that study, about 70% of cases presented mucosae thickness ≤ 3 mm, while in the present study, 78% of the mucosae had a thickness ≤ 3 mm. Moreover, it has to be considered that the thickness of the sinus mucosa is different in various regions of the sinus [19].

In the present study, the virtual thickness of the sinus mucosa increased by 5 mm after 7 days from surgery, and the virtual area increased by 147.3 mm^2^. After 9 months, both virtual mucosa thickness and area regressed to values similar to or lower than those observed at the baseline. These outcomes are in agreement with a series of experimental and clinical studies [4–8].

On the radiographic evaluation, it was difficult to differentiate the sinus membrane from the subjacent sub-mucosal edema and bleeding that both contributed to a transient thickening and additional elevation of the sinus mucosa (transient mucosal thickening) [6, 8]. For this reason, in the present study, it was introduced the term “virtual” when referring to sinus mucosa thickness and area. Experimental [5] and clinical studies [15, 16] reported data of the sinus mucosa thickening after sinus floor augmentation. After 5 days of healing from sinus floor elevation in monkeys [5], both macro and microscopically were seen residues of clot filling the elevated space. In a clinical study [15], fifty-three CBCTs were assessed in patients treated for sinus floor augmentation. The sinus mucosa showed an increase in volume immediately after surgery and a normalization of the volumes after 7.5 months on average. In another clinical study [16], thirty-two maxillary sinus floor augmentations were performed and the CBCTs were taken before surgery and at different times afterward. The thickness before surgery was 0.75 mm and gradually increased to 2.36 mm, 4.14 mm, 6.05 mm, and 6.63 mm, respectively after 1, 2, 3, and 7 days. At the 6-month follow-up, the thickness returned to normality. Another clinical study [7], the evaluation of CBCTs taken before sinus floor augmentation and after 1 week and 3 months showed that the edema was present in all augmented sinus after 1 week and that the mucosa thickness returned to normality in 96% of cases after 3 months.

The post-operative edema and bleeding that increase the mucosa thickness might also reach the region of the MSO and causing its obstruction and a possible impairment drainage capacity of sinus mucus. The reduction of the patency and obstruction of the MSO and infundibulum can lead to inflammatory and/or infectious processes of the sinus cavity, causing acute or chronic sinusitis [20–22].

In the present study, the MSO was located at an average distance of about 33.3 mm from the sinus floor, reporting a minimum value of 25.2 mm in one case, a distance far away from the areas interested by the floor elevation procedure. However, the increased transient mucosa thickness must be considered, especially at the palatal aspect of the sinus. In the present study, the mean distance between MSO and the highest extension of the edematous mucosa at the palatal aspect was 10.3 mm, so still far away from the MSO. However, when the data were evaluated individually, in eight cases, the edema reached the MSO.

The mean diameter of the MSO before surgery was 1.8 mm that was found reduced to 1.1 mm after 1 week. However, when the data were evaluated individually, in thirteen cases, the diameter of the MSO was = 0 and in twenty-six cases < 1 mm. Three cases presented a loss of patency of the infundibulum before sinus floor augmentation, while 14 were detected after 1 week of healing, including two of the three infundibula that were already in such conditions before surgery. However, after 9 months of healing, the mean diameter of the MSO was 1.5 mm, and only one infundibulum was out of patency, a condition already exhibited after 1 week, but absent before surgery. Nevertheless, no signs of sinus pathology were detected radiographically and clinically after 9 months, and no complaints were reported by the patient. Moreover, the sinus mucosa presented lower thickness and area compared to the initial stage.

Data regarding the MSO involvement after sinus floor augmentation were reported in a prospective cohort study that evaluated the membrane thickness and ostium patency following sinus floor augmentation [15]. Fifty-three patients were included in that study. The initial sinus mucosa thickness was 1.9 mm (range 0.47 mm; 8.42 mm), and 7 MSO (13%) were found obstructed before sinus floor elevation. Immediately after surgery, the swelling of the sinus mucosa increased the thickness to 4.07 mm and 16 (30%) obstructions were detected. After a mean of 7.5 months, the sinus mucosa thickness regressed to 1.9 mm and 5 (9.4%) obstructions were still observed.

The three obstructions of the infundibula found in the present study were all associated with a thickening of the virtual sinus mucosa. Two of these obstructions presented a mucus retention cyst that was drained at the surgical session. A retrospective study on 310 maxillary sinuses evaluated in 156 CBCTs reported a frequency of 12.9% of mucus retention cysts, of which only 28.6% were found on the floor of the sinus [23]. In the other obstruction reported in the present study, no cysts could be detected and no connections with other pathologies could be associated, such as allergies or heavy smoke. These patients with an initial obstruction of the infundibula were partially dentate so that the thickening of the Schneiderian mucosa could be ascribed to some dental pathologies suffered by the patients.

In the present study, seven accessory maxillary ostia (AMO) were detected, reaching a fraction of 9.7% of the total sinuses evaluated. This percentage was lower than that reported by another tomographic study (17.9%) in which younger patients were included compared to the present study [22]. In that study, it was shown that patient age and status of dentition were influencing factors on the number of AMOs. The mean distance of the accessory ostia from the *X*-axis was 7.8 mm, closer to the *X*-axis compared to the primary MSO.

Six sinus mucosae were perforated in the present study during surgery, and they were protected with a collagen membrane. No obliteration of the MSO and infundibulum were detected at any period of evaluation in those cases, and the sinus mucosa was thinner in all periods evaluated compared to the mean values of all sinuses evaluated.

As an exploratory aim, the data were categorized according to gender and type of edentulism (Table 5). A stronger edema reaction in the sinus mucosa was observed in male compared to female patients. It is interesting to note that this higher width of the sinus mucosa in males was mainly due to ten sinuses in nine patients that presented a baseline mean thickness of 2.1 mm that increased after 1 week to a mean of 21.2 mm. Conversely, the remaining male patients presented a mean baseline virtual thickness of 2.6 mm that increased to 4.0 mm after 1 week of healing. The difference in virtual mucosa thickness after 1 week might be ascribed to different post-surgical bleeding in the subantral space in the two groups of male patients. Nevertheless, further studies should be performed to evaluate possible correlations between the post-surgery virtual thickness of the mucosa and anatomical structures, nasal pathologies, and systemic conditions.

Moreover, after 9 months of healing, a restoration of a normal ostium diameter was observed in males, while female patients presented a loss of ~ 22% of the diameter.

At the baseline, the sinus mucosa was thicker at the partial compared to the fully edentulous patients. This is in agreement with a retrospective study in which 338 sinuses in 169 patients were evaluated in CBCTs [24]. It was found that a thickening of the sinus mucosa, defined as radiodensity, was present in 16.6% of patients and 10.4% sinuses. Moreover, the proportions of these “radiodensities” were higher at the dentate (62.9%) compared to the partially edentulous (34.2%) and the full edentulous (2.9%) patients. It can be argued that in the partially edentulous patients, apical or periodontal pathologies in the distal maxillary segments might be still present. These pathologies might affect the sinus mucosa thickness [25–27]. Moreover, it has been shown that the treatment of apical lesions [27] or the extraction of teeth with pathologies [25, 28] might reduce the sinus mucosa thickness over time.

However, after 9 months, the reduction in sinus mucosa thickness was higher at the partial compared to the fully edentulous patients. The ostium diameter was similar in both groups in all periods examined.

From a clinical point of view, before sinus lifting, it is important to evaluate not only the presence of sinusal pathologies but also, among the various risk factors considered [29], the patency of the ostium to consent a proper sinusal drainage. CBCTs including the full length of the infundibulum, from the ostium to the hiatus semilunaris, should be performed to allow a correct evaluation of the patency of the entire sinusal drainage tract. Moreover, due to the transient edema of the sinus mucosa and the reduction of the patency of the ostium after sinus lifting, a strict monitoring of the general and local health conditions is recommended to disclose as earlier as possible the signs of complications such as sinusitis.

As limitations of the present retrospective analysis, it should be considered that all patients were not smokers and that 3D data were not available. Moreover, CBCT with a higher image definition should be performed to obtain more accurate data.

In conclusion, 1 week after sinus floor augmentation, the maxillary sinus mucosa increased in dimensions and in several cases involved the ostium, reducing its diameter and producing a transient loss of patency. After 9 months of healing, the initial conditions were recovered.

## Data Availability

The datasets used or analyzed during the current study are available from the corresponding author on reasonable request.

## References

[1] Boyne PJ, James RA (1980). Grafting of the maxillary sinus floor with autogenous marrow and bone. J Oral Surg..

[2] Del Fabbro M, Wallace SS, Testori T (2013). Long-term implant survival in the grafted maxillary sinus: a systematic review. Int J Periodontics Restorative Dent..

[3] Cavalcanti MC, Guirado TE, Sapata VM, Costa C, Pannuti CM, Jung RE, César Neto JB (2018). Maxillary sinus floor pneumatization and alveolar ridge resorption after tooth loss: a cross-sectional study. Braz Oral Res..

[4] Scala A, Botticelli D, Rangel IG, de Oliveira JA, Okamoto R, Lang NP (2010). Early healing after elevation of the maxillary sinus floor applying a lateral access: a histological study in monkeys. Clin Oral Implants Res..

[5] Scala A, Botticelli D, Faeda RS, Garcia Rangel I, Américo de Oliveira J, Lang NP (2012). Lack of influence of the Schneiderian membrane in forming new bone apical to implants simultaneously installed with sinus floor elevation: an experimental study in monkeys. Clin Oral Implants Res..

[6] Quirynen M, Lefever D, Hellings P, Jacobs R (2014). Transient swelling of the Schneiderian membrane after transversal sinus augmentation: a pilot study. Clin Oral Implants Res..

[7] Nosaka Y, Nosaka H, Arai Y (2015). Complications of postoperative swelling of the maxillary sinus membrane after sinus floor augmentation. J Oral Sci Rehabil.

[8] Temmerman A, Van Dessel J, Cortellini S, Jacobs R, Teughels W, Quirynen M (2017). Volumetric changes of grafted volumes and the Schneiderian membrane after transcrestal and lateral sinus floor elevation procedures: a clinical, pilot study. J Clin Periodontol..

[9] Kawakami S, Lang NP, Iida T, Ferri M, Apaza Alccayhuaman KA, Botticelli D (2018). Influence of the position of the antrostomy in sinus floor elevation assessed with cone-beam computed tomography: a randomized clinical trial. J Investig Clin Dent..

[10] Kawakami S, Lang NP, Ferri M, Apaza Alccayhuaman KA, Botticelli D. Influence of the height of the antrostomy in sinus floor elevation assessed by cone beam computed tomography - a randomized clinical trial. Int J Oral Maxillofac Implants. 2019 January/February;34(1):223–232. 10.11607/jomi.7112 30521653

[11] Hirota A, Lang NP, Ferri M, Fortich Mesa N, Apaza Alccayhuaman KA (2019). Botticelli Tomographic evaluation of the influence of the placement of a collagen membrane subjacent to the sinus mucosa during maxillary sinus floor augmentation: a randomized clinical trial. Int J Implant Dent..

[12] Timmenga NM, Raghoebar GM, Liem RS, van Weissenbruch R, Manson WL, Vissink A (2003). Effects of maxillary sinus floor elevation surgery on maxillary sinus physiology. Eur J Oral Sci..

[13] Griffa A, Berrone M, Boffano P, Viterbo S, Berrone S (2010). Mucociliary function during maxillary sinus floor elevation. J Craniofac Surg..

[14] Torretta S, Mantovani M, Pignataro L (2012). In reply to “mucociliary function during maxillary sinus floor elevation”. J Craniofac Surg..

[15] Guo ZZ, Liu Y, Qin L, Song YL, Xie C, Li DH (2016). Longitudinal response of membrane thickness and ostium patency following sinus floor elevation: a prospective cohort study. Clin Oral Implants Res..

[16] Makary C, Rebaudi A, Menhall A, Naaman N (2016). Changes in sinus membrane thickness after lateral sinus floor elevation: a radiographic study. Int J Oral Maxillofac Implants..

[17] Kawakami S, Botticelli D, Nakajima Y, Sakuma S, Baba S (2019). Anatomical analyses for maxillary sinus floor augmentation with a lateral approach: a cone beam computed tomography study. Ann Anat..

[18] Lozano-Carrascal N, Salomó-Coll O, Gehrke SA, Calvo-Guirado JL, Hernández-Alfaro F, Gargallo-Albiol J (2017). Radiological evaluation of maxillary Sinus anatomy: a cross-sectional study of 300 patients. Ann Anat..

[19] Janner SF, Caversaccio MD, Dubach P, Sendi P, Buser D, Bornstein MM (2011). Characteristics and dimensions of the Schneiderian membrane: a radiographic analysis using cone beam computed tomography in patients referred for dental implant surgery in the posterior maxilla. Clin Oral Implants Res..

[20] de Carvalho ABG, Ferreira Costa AL, Fuziy A, de Assis ACS, Castro Veloso JR, Coutinho Manhães LR, Santamaria MP, de Castro Lopes SLP (2018). Investigation on the relationship of dimensions of the maxillary sinus drainage system with the presence of sinusopathies: a cone beam computed tomography study. Arch Oral Biol.

[21] Shanbhag S, Shanbhag V, Stavropoulos A (2014). Volume changes of maxillary sinus augmentations over time: a systematic review. Int J Oral Maxillofac Implants..

[22] Yeung AWK, Colsoul N, Montalvao C, Hung K, Jacobs R, Bornstein MM. Visibility, location, and morphology of the primary maxillary sinus ostium and presence of accessory ostia: a retrospective analysis using cone beam computed tomography (CBCT). Clin Oral Investig. 2019;9. 10.1007/s00784-019-02829-9 30737619

[23] Yeung AWK, Tanaka R, Khong PL, von Arx T, Bornstein MM (2018). Frequency, location, and association with dental pathology of mucous retention cysts in the maxillary sinus. A radiographic study using cone beam computed tomography (CBCT). Clin Oral Investig..

[24] Kawai T, Tanaka R, Yeung AWK, von Arx T, Bornstein MM (2019). Frequency and type of incidentally detected radiodensities in the maxillary sinus: a retrospective analysis using cone beam computed tomography (CBCT). Clin Oral Investig..

[25] Yoo JY, Pi SH, Kim YS, Jeong SN, You HK (2011). Healing pattern of the mucous membrane after tooth extraction in the maxillary sinus. J Periodontal Implant Sci..

[26] Vogiatzi T, Kloukos D, Scarfe WC, Bornstein MM (2014). Incidence of anatomical variations and disease of the maxillary sinuses as identified by cone beam computed tomography: a systematic review. Int J Oral Maxillofac Implants..

[27] Lu Y, Liu Z, Zhang L, Zhou X, Zheng Q, Duan X, Zheng G, Wang H, Huang D (2012). Associations between maxillary sinus mucosal thickening and apical periodontitis using cone-beam computed tomography scanning: a retrospective study. J Endod..

[28] Nurbakhsh B, Friedman S, Kulkarni GV, Basrani B, Lam E (2011). Resolution of maxillary sinus mucositis after endodontic treatment of maxillary teeth with apical periodontitis: a cone-beam computed tomography pilot study. J Endod..

[29] Testori T, Yu SH, Tavelli L, Wang HL. Perforation risk assessment in maxillary sinus augmentation with lateral wall technique. Int J Periodontics Restorative Dent. 2020 May/Jun;40(3):373-380. doi: 10.11607/prd.4179. 10.11607/prd.4179 32233190

